# Report of the Committee of Distribution to the Council

**Published:** 1914-08-08

**Authors:** 


### REPORT OF THE COMMITTEE OF DISTRIBUTION TO THE COUNCIL.

The Committee of Distribution held their first meeting on Tuesday, May 26, and then and at subsequent meetings examined the reports and income and expenditure accounts of the several institutions seeking to participate, and made their awards in conformity with the Laws of the Constitution.

The Committee beg to report that the total of the Fund on August 5 will amount to £59,500.

The Fund has received a legacy of £100 1s. 1d. from the estate of the Rev. W. West, deceased.

This year 253 institutions have applied to participate in the Fund, being ten less than in 1913, and the Committee now recommend the distribution of £59,403 to the several hospitals, institutions, dispensaries, and nursing associations as shown in the appendix hereto.

The Committee having reduced the bases of award to seven institutions, the governing bodies of the same were informed thereof in accordance with the Laws of the Constitution. Four deputations attended the Committee, and after hearing their several explanations, the bases of award were finally determined.

Seven and a half per cent. of the total sum available for distribution is appropriated to the purchase of surgical appliances during the ensuing year, and 2½ per cent. for district nursing associations.

In compliance with an order of the Council, statistics have been prepared as usual for the special use of its members, showing the number of beds in hospitals, the cost of patients at hospitals and dispensaries, the proportionate expense of management to maintenance, and other valuable information. A copy thereof has been sent to each member of the Council.

T. Vansittart Bowater, Lord Mayor, President and Treasurer.

David Anderson. G. H. Makins.

John C. Bell. Adrian Pollock.

F. A. Bevan. Marcus Samuel.

William S. Church. E. Parker Young.

Savile Crossley.

Richard B. Martin, } Hon.
John Henry Buxton, } Secs.

The Mansion House, July 23, 1914.

---

### Awards Recommended by the Committee of Distribution for the Year 1914.

### THIRTY-THREE GENERAL HOSPITALS.

Charing Cross Hospital, £1,080; French Hospital, £356 1s. 3d.; German Hospital, £600 15s.; Great Northern Central Hospital, £1,298 10s. 6d.; Guy's Hospital, £1,692 11s. 3d.; Hampstead and N.W. London Hospital, £712 19s. 4d.; Italian Hospital, £205 0s. 6d.; Kensington General Hospital, £116 8s. 9d.; King's College Hospital, £1,454 5s.; London Hospital, £5,707 19s. 4d.; London Homœopathic Hospital, £507 18s. 9d.; Phillips' Memorial Homœopathic Hospital, £43 17s. 6d.; London Temperance Hospital, £608 6s. 10d.; Metropolitan Hospital, £1,079 3s. 2d.; Mildmay Hospital, £226 2s. 6d.; Mildmay Memorial Hospital, £49 15s. 8d.; Miller Hospital and Royal Kent Dispensary, £389 16s. 3d.; Poplar Hospital, £557 14s. 4d.; Prince of Wales's General Hospital, Tottenham, £790 11s. 10d.; Royal Free Hospital, £1,276 11s. 10d.; St. George's Hospital, £2,041 0s. 7d.; SS. John and Elizabeth Hospital, £148 10s.; St. John's Hospital, Lewisham, £221 3s. 5d.; St. Mary's Hospital, £1,875 13s. 1d.; St. Thomas's Hospital, £478 8s. 2d.; Seamen's Hospital Society, £1,400 12s. 6d.; the Middlesex Hospital and Convalescent Home, £2,465 8s. 9d.; University College Hospital, £1,830 1s. 10d.; Walthamstow, etc., Hospital, £140 18s. 1d.; Wandsworth, Bolingbroke Hospital, £317 10s.; West Ham Hospital, £800 6s.; West London Hospital, £1,131 4s. 4d.; Westminster Hospital, £1,003 4s. 4d.

### SIX CHEST HOSPITALS.

City of London Hospital for Diseases of the Chest, Victoria Park, £888 5s. 3d.; Hospital for Consumption, Brompton, £2,213 3s. 2d.; Mount Vernon Consumption Hospital, Hampstead, £779 12s. 6d.; Royal Hospital for Diseases of the Chest, £507 1s. 10d.; Royal National Sanatorium, £67 10s.; Royal National Hospital for Consumption, £210 18s. 9d.

### TWENTY CHILDREN'S HOSPITALS.

Alexandra Hospital for Hip Disease, £289 8s. 2d.; Banstead Surgical Home, £20 5s.; Barnet Home Hospital, £32 18s. 2d.; Belgrave Hospital for Children, £210 1s. 10d.; Cheyne Hospital for Incurable Children, £135 17s.; East London Hospital for Children, £820 2s. 6d.; Evelina Hospital for Sick Children, £201 13s. 2d.; Home for Incurable Children, £41 6s. 10d.; Home for Sick Children, Sydenham, £102 2s.; Hospital for Sick Children, £1,228 10s.; Infants' Hospital, Westminster, £199 2s. 6d.; Kensington, for Children and Dispensing, £81; Paddington Green Hospital for Children, £243; Queen's Hospital for Children, £843 15s.; Royal Waterloo Hospital for Children and Women, £477 11s. 3d.; St. Mary's Hospital, Plaistow, £250 11s. 10d.; St. Monica's Hospital, Brondesbury, £66 13s. 2d.; Victoria Hospital for Children, Chelsea, £613 8s. 2d.; Victoria Home, Margate, £39 13s. 2d.; Hospital for Hip Disease, Sevenoaks, £54 16s. 10d.

### SEVEN LYING-IN HOSPITALS.

City of London Lying-in Hospital, £156 1s. 10d.; Clapham Maternity Hospital and Dispensary, £28 13s. 9d.; East End Mothers' Home, £70 17s. 6d.; General Lying-in Hospital, Lambeth, £202 10s.; Plaistow Maternity Hospital, £45 11s. 3d.; Queen Charlotte's Lying-in Hospital, £491 18s. 2d.; Home for Mothers and Babies, Woolwich, £44 14s. 4d.

### FIVE HOSPITALS FOR WOMEN.

Chelsea Hospital for Women, £287 14s. 4d.; Hospital for Women, Soho Square, £377 3s. 2d.; Grosvenor Hospital for Women and Children, £122 7s.; New Hospital for Women, £313 0s. 6d.; Samaritan Free Hospital, £394 17s. 6d.

### TWENTY-FIVE OTHER SPECIAL HOSPITALS.

Middlesex Hospital, Cancer Wing, £81 16s. 10d.; London Fever Hospital, Islington, £253 2s. 6d.; Gordon Hospital for Fistula, £25 6s. 3d.; St. Mark's Hospital for Fistula, £315 11s. 3d.; National Hospital for the Diseases of the Heart, £94 10s.; Female Lock Hospital, Harrow Road, £318 2s.; Hospital for Epilepsy, Paralysis, and other Diseases of the Nervous System, £171 5s. 8d.; National Hospital for the Paralysed and Epileptic, £808 6s. 3d.; West End Hospital for Diseases of the Nervous System, £310 10s.; Central London Ophthalmic Hospital, £147 13s. 2d.; Royal Eye Hospital, £226 2s. 6d.; Royal London Ophthalmic Hospital, £837 17s.; Royal Westminster Ophthalmic Hospital, £138 7s. 6d.; Western Ophthalmic Hospital, £65 16s. 3d.; Royal National Orthopædic Hospital, £567 16s. 10d.; Royal Sea Bathing Hospital, Margate, £120 13s.; St. John's Hospital for Diseases of the Skin, £123 3s. 9d.; St. Peter's Hospital for Stone, £120 13s.; Central London Throat and Ear Hospital, £35 8s. 9d.; Throat Hospital, Golden Square, £44 14s. 4d.; London Throat Hospital, £30 7s. 6d.; Metropolitan Ear, Nose, and Throat Hospital, £42 3s. 9d.; Royal Ear Hospital, Dean Street, £83 10s. 6d.; Royal Dental Hospital of London, £141 15s.; National Dental Hospital, £61 12s.

### THIRTY-THREE CONVALESCENT HOSPITALS.

Metropolitan Convalescent Institution, Walton, £308 16s. 3d.; Metropolitan Convalescent Institution, Broadstairs, £227 16s. 3d.; Metropolitan Convalescent Institution, Bexhill, £433 13s. 9d.; All Saints' Convalescent Hospital, Eastbourne, £337 10s.; All Saints' Convalescent Home, St. Leonards, £21 18s. 9d.; Ascot Priory Convalescent Home, £67 10s.; Brentwood Convalescent Home for Children, £14 6s. 10d.; Charing Cross Hospital Convalescent Home, £42 3s. 9d.; Chelsea Hospital for Women Convalescent Home, St. Leonards, £41 6s. 10d.; Deptford Medical Mission Convalescent Home, £15 3s. 9d.; Mrs. Gladstone's Convalescent Home, Mitcham, £67 10s.; Friendly Societies' Convalescent Home, Dover, £84 7s. 6d.; Hahnemann Convalescent Home, Bournemouth, £21 1s. 10d.; Hanwell Convalescent Home, £6 15s.; Hastings, Fairlight Convalescent Home, £32 1s. 3d.; Herbert Convalescent Home, Bournemouth,

£14 6s. 10d.; Herne Bay, Baldwin-Brown Convalescent Home, £50 12s. 6d.; Homœopathic Hospital Convalescent Home, £4 4s. 6d.; Hemel Hempstead Convalescent Home, £21 18s. 9d.; Mrs. Kitto's Convalescent Home, Reigate, £39 13s.; London Hospital Convalescent Home, Tankerton, £63 5s. 7d.; Police Seaside Home, Brighton, £37 19s. 4d.; St. Andrew's Convalescent Home, Clewer, £75 18s. 9d.; St. Andrew's Convalescent Home, Folkestone, £135; St. John's Home for Convalescent and Crippled Children, £16 0s. 6d.; St. Joseph's Convalescent Home, Bournemouth, £21 18s. 9d.; St. Leonards-on-Sea Convalescent Home for Poor Children, £91 19s. 4d.; Eversfield, St. Leonard's, £25 6s. 3d.; St. Mary's Home, Birchington, £21 2s.; St. Mary's Convalescent Home, Broadstairs, £126 11s. 3d.; St. Mary's Convalescent Home, Shortlands, £15 3s. 9d.; St. Michael's Convalescent Home, Westgate-on-Sea, £27 16s. 10d.; Seaside Convalescent Hospital, Seaford, £71 14s. 4d.

### TWENTY-FOUR COTTAGE HOSPITALS.

Acton Cottage Hospital, £112 4s. 4d.; Beckenham Cottage Hospital, £79 6s. 3d.; Blackheath and Charlton Cottage Hospital, £80 3s. 2d.; Bromley, Kent, Cottage Hospital, £89 8s. 9d.; Bushey Heath Cottage Hospital, £29 10s. 7d.; Canning Town Cottage Hospital, £86 1s. 3d.; Chislehurst, Sidcup, and Cray Cottage Hospital, £56 11s. 3d.; Coldash Cottage Hospital, £47 5s.; Ealing Cottage Hospital, £129 18s. 9d.; East Ham Cottage Hospital, £56 10s. 6d.; Eltham Cottage Hospital, £49 15s. 6d.; Enfield Cottage Hospital, £78 9s. 4d.; Epsom and Ewell Cottage Hospital, £41 6s. 10d.; Hounslow Cottage Hospital, £34 4s.; Livingstone Dartford Cottage Hospital, £99 2s. 6d.; Kingston, Victoria Hospital, £46 10s. 11d.; Reigate and Redhill Hospital, £143 2s. 2d.; Sidcup Cottage Hospital, £24 5s. 6d.; Teddington, £54 8s. 9d.; Tilbury (Passmore Edwards) Cottage Hospital, £58 3s. 8d.; Willesden Cottage Hospital, £86 1s. 3d.; Wimbledon Cottage Hospital, £64 2s. 6d.; Wimbledon Nelson Hospital, £56 18s. 2d.; Wood Green Cottage Hospital, £69 9s. 9d.

### ELEVEN INSTITUTIONS.

Florence Nightingale Hospital for Invalid Gentlewomen, £86 18s. 1d.; St. Saviour's Hospital and Nursing Home, £65 16s. 2d.; Invalid Asylum, Stoke Newington, £27 16s. 10d.; Firs Home, Bournemouth, £29 10s. 7d.; Friedenheim Hospital for the Dying, £250 11s. 10d.; Free Home for Dying, Clapham, £129 18s. 9d.; St. Luke's House, Pembridge Square, £128 5s.; Santa Claus Home, Highgate, £64 2s. 6d.; Royal Mineral Water Hospital, Bath, £50 12s. 6d.; Winifred House, Holloway, £48 18s. 9d.; Highgate Convalescent Home, £37 2s. 6d.

### FIFTY-SIX DISPENSARIES.

Battersea Provident Dispensary, £163 13s. 9d.; Billingsgate Dispensary, £45 11s. 3d.; Blackfriars Provident Dispensary, £15 3s. 9d.; Bloomsbury Provident Dispensary, £12 13s.; Brixton, etc., Dispensary, £40 10s.; Brompton Provident Dispensary, £15 3s. 9d.; Buxton Street Dispensary, £9 5s. 6d.; Camberwell Provident Dispensary, £49 15s. 7d.; Chelsea Provident Dispensary, £10 2s. 6d.; Child's Hill Provident Dispensary, £6 15s.; City Dispensary, £58 4s. 4d.; Clapham General and Provident Dispensary, £18 11s.; Deptford Medical Mission, £23 12s. 6d.; Eastern Dispensary, £30 7s. 6d.; East Dulwich Provident Dispensary, £50 12s. 6d.; Farringdon General Dispensary, £34 12s.; Finsbury Dispensary, £43 17s. 6d.; Forest Hill Provident Dispensary, £32 18s.; Greenwich Provident Dispensary, £23 12s. 6d.; Hackney Provident Dispensary, £16 0s. 6d.; Hampstead Provident Dispensary, £37 2s. 6d.; Holloway and North Islington Dispensary, £12 13s.; Islington Dispensary, £39 13s.; Islington Medical Mission, £46 13s.; Kennington and Vauxhall Provident Dispensary, £11 16s. 3d.; Kensal Town Provident Dispensary, £12 13s.; Kentish Town Medical Mission, £18 11s.; Kilburn, Maida Vale, and St. John's Wood Dispensary, £28 13s. 9d.; Kilburn Provident Medical Institution, £39 13s.; London Dispensary, Spitalfields, £10 19s. 4d.; London Medical Mission, Endell Street, £73 8s.; Margaret Street, for Consumption, £22 15s. 8d.; Metropolitan Dispensary, £38 16s. 3d.; Mildmay Medical Mission Dispensary, £8 8s. 9d.; Notting Hill Provident Dispensary, £19 8s. 1d.; Paddington Provident Dispensary, £29 10s. 7d.; Public Dispensary, Drury Lane, W.C., £32 18s.; Queen Adelaide's Dispensary, £17 14s. 4d.; Royal General Dispensary, £21 1s. 10d.; Royal Pimlico Provident Dispensary, £21 18s. 8d.; Royal South London Dispensary, £36 5s. 7d.; St. George's, Hanover Square, Dispensary, £27 16s. 10d.; St. John's Wood Provident Dispensary, £39 13s.; St. Marylebone General Dispensary, £54; St. Pancras and Northern Dispensary, £37 2s. 6d.; South Lambeth, Stockwell, and North Brixton Dispensary, £17 14s. 4d.; Stamford Hill, Stoke Newington Dispensary, £46 7s. 9d.; Tower Hamlets Dispensary, £32 18s.; Walworth Provident Dispensary, £11 16s. 3d.; Wandsworth Common Provident Dispensary, £7 11s. 10d.; Westbourne Provident Dispensary, £14 6s. 10d.; Western Dispensary, £56 10s. 6d.; Western General Dispensary, £70 17s. 6d.; Westminster General Dispensary, £32 1s.; Whitechapel Provident Dispensary, £23 12s. 6d.; Woolwich Provident Dispensary, £29 10s. 7d.

### THIRTY-TWO NURSING ASSOCIATIONS.

Belvedere, Abbey Wood, £5; Brixton, £25; Central St. Pancras, £20; Chelsea and Pimlico, £20; Hackney, £25; Hammersmith, £50; Hampstead, £15; Isleworth, £10; Kensington, £40; Kilburn, £10; Kingston, £35; Lambeth Road (Catholic), £10; Metropolitan (Bloomsbury), £35; St. Olave's (Bermondsey), £20; Paddington and Marylebone, £45; Plaistow, £115; Plaistow (Maternity), £135; Ponders End, Enfield, etc., £10; Rotherhithe, £10; Shoreditch, £45; Sick Room Helps Society, £20; Sidcup, £5; Silvertown, £15; South London (Battersea), £45; Southwark, £40; South Wimbledon, £25; Tottenham, £20; Westminster, £20; Woolwich, £30; East London, £120; North London, £50; Ranyard Nurses, £350.

The Council are prepared to receive and distribute as part of the annual receipts special gifts or bequests from any who may wish to assist the hospitals of London and are in doubt as to those most deserving their bounty.

Grants are made only to institutions furnishing the Committee of Distribution with proofs of their usefulness and efficiency and properly audited accounts.

